# Form follows function: Nuclear morphology as a quantifiable predictor of cellular senescence

**DOI:** 10.1111/acel.14012

**Published:** 2023-10-16

**Authors:** Jakub Belhadj, Surina Surina, Markus Hengstschläger, Alexis J. Lomakin

**Affiliations:** ^1^ Center for Pathobiochemistry & Genetics, Institute of Medical Genetics Medical University of Vienna Vienna Austria; ^2^ Center for Pathobiochemistry & Genetics, Institute of Medical Chemistry and Pathobiochemistry Medical University of Vienna Vienna Austria; ^3^ School of Medical Sciences University of Campania Luigi Vanvitelli Napoli Italy

**Keywords:** artificial intelligence, cell nucleus, cellular biophysics, cellular senescence, computer vision, machine learning, morphogenesis, quantitative microscopy

## Abstract

Enlarged or irregularly shaped nuclei are frequently observed in tissue cells undergoing senescence. However, it remained unclear whether this peculiar morphology is a cause or a consequence of senescence and how informative it is in distinguishing between proliferative and senescent cells. Recent research reveals that nuclear morphology can act as a predictive biomarker of senescence, suggesting an active role for the nucleus in driving senescence phenotypes. By employing deep learning algorithms to analyze nuclear morphology, accurate classification of cells as proliferative or senescent is achievable across various cell types and species both in vitro and in vivo. This quantitative imaging‐based approach can be employed to establish links between senescence burden and clinical data, aiding in the understanding of age‐related diseases, as well as assisting in disease prognosis and treatment response.

## Graphical abstract

Nuclear morphology (size and shape) is a quantifiable marker of senescence. Deep learning algorithms, detecting and measuring nuclear morphology, consistently classify senescent and proliferating cells across various cell types and species, holding clinical potential. Experimental manipulation of nuclear size can trigger a senescence (‐like) state, confirming the predictive power of nuclear morphology. A causal link between nuclear morphology and senescence warrants further research.

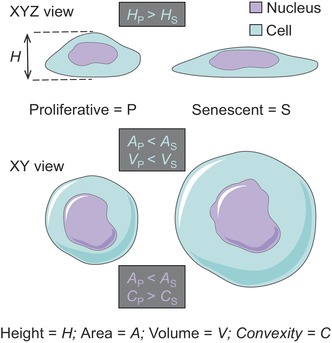

**Abbreviations**
DNNs: deep neural networks
DTX: docetaxel
GSEA: gene set enrichment analysis
H&E: hematoxylin and eosin
SAMPs: senescence‐associated morphological profiles
SASP: senescence‐associated secretory phenotype
SA‐β‐gal: senescence‐associated β‐galactosidase
WGD: whole‐genome duplication

“Form follows function” is a design principle widely utilized in architecture (Guillén, 2020), asserting that extensive physical properties of an object, such as shape and size, should primarily correspond to its intended function. This axiom can similarly be extended to biological form across various scales of organization, encompassing cells (Lomakin et al., 2015), subcellular organelles (Wang & Youle, 2016), and macromolecular complexes (Kosak & Groudine, 2004). One example of this is the morphogenetic change that tissue cells experience during senescence (from Latin *senēscere* “to grow old”) (Figure 1). Cellular senescence is a state of persistent proliferative arrest that cells enter in response to stressors or as a result of biological aging (Gorgoulis et al., 2019). While senescent cells cease proliferation, they continue being metabolically active (albeit in an altered manner) and synthesize a plethora of bioactive compounds contributing to the aging process (Gorgoulis et al., 2019). The proliferative block and thus inability to mitotically partition the material accumulated via biosynthetic activity might contribute to cell size enlargement. However, in the context of senescence, cellular hypertrophy is likely attributed to deficiencies within the cellular degradation system, exemplified by impaired lysosomal function (Rovira et al., 2022).

**FIGURE 1:**
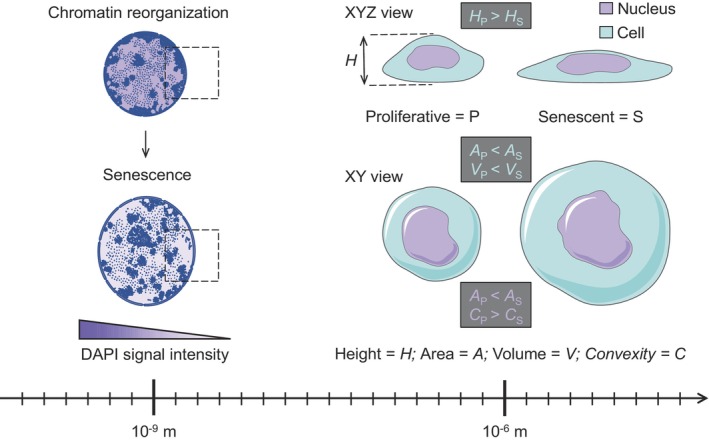
Observable and quantifiable manifestations of cellular senescence across different scales of organization including whole‐cell level, the nucleus, and nuclear chromatin.

In addition to the mere volume increase, which is a cell size change, senescent cells reinforce and remodel their cytoskeletal elements physically forcing the cells to flatten (Brauer et al., 2023), which is a cell shape change. Cell flattening co‐contributes to the apparent cell size change because it increases projected cell surface area, the most noticeable phenotypic trait of senescent cells under a microscope (Gorgoulis et al., 2019). The cell size increase per se can be both a strong driver and a reinforcing mechanism of senescence. This is because cytoplasmic volume expansion beyond a threshold level (set by an optimal nucleocytoplasmic ratio) may result in the physicochemical effect of cytoplasm dilution in which the concentration of critical molecular regulators responsible for DNA replication‐reparation and cell cycle progression gets significantly decreased (Lanz et al., 2022; Neurohr et al., 2019). This in turn ensures that while “growing old,” senescent cells do not engage molecular programs associated with physiologically normal proliferation. At the same time, the enlarged and flattened morphology of senescent cells (resulting in the increased projected cell surface area) is expected to allow for enhanced secretion of extracellular signals and thus communication with neighboring cells. This should facilitate the establishment of the senescence‐associated secretory phenotype (SASP), a biochemical hallmark of cellular senescence (Gorgoulis et al., 2019).

The nucleus is the largest intracellular compartment sensitive to cell flattening. When a cell is flattened below a resting nuclear diameter either artificially via compression‐stretching or physiologically due to enhanced spreading on the extracellular matrix, its nucleus deforms and acquires a flattened morphology (Lomakin et al., 2020). The latter manifests itself as increased projected nuclear surface area, yet another hallmark of senescence (Mitsui & Schneider, 1976).

Nuclear flattening may trigger mechanosensitive calcium release, alterations in lipid signaling and metabolism, chromatin reorganization, and even DNA damage (Lomakin et al., 2020; Nava et al., 2020; Shah et al., 2021). When the nucleus is flattened and thus compressively expanded beyond its membrane areal capacity, it ruptures (Srivastava et al., 2021). Nuclear rupture leads to the leakage of the nucleoplasm into the cytoplasmic domain diluting the intranuclear concentration of DNA replication‐reparation factors (Xia et al., 2018). At the same time, the breach of the nucleocytoplasmic barrier exposes nuclear DNA to the molecular sensors of cytosolic DNA able to initiate proinflammatory cell signaling (Nader et al., 2021). All these processes downstream of nuclear flattening and compressive expansion are also considered hallmarks of senescent cells (Criscione et al., 2016; Gluck et al., 2017; Hewitt et al., 2012; Mu et al., 2020; Wiley et al., 2021; Ziegler et al., 2021). The causal link between nuclear flattening per se and the induction of cellular senescence has been demonstrated in reductionist settings in which isolated cells were flattened below their resting nuclear diameter in highly controlled and calibrated microfabricated devices (Nader et al., 2021).

These examples illustrate that specific molecular‐scale events (e.g., signaling and metabolic alterations) driving senescence can be encoded in the observable cell morphology. This in turn suggests that assessing cellular and subcellular shape and size might have predictive power in decoding a senescent cell state using relatively straightforward analytical and quantitative imaging approaches (Kusumoto et al., 2021). It is therefore not surprising that the stereotypic changes in cell and nuclear morphology during senescence have been recently classified as critical components of senescence‐associated morphological profiles (SAMPs). These profiles are proposed as robust “first‐pass” tools facilitating the distinction between senescent and proliferating cells to streamline experimental workflows and complement conventional markers of senescence and senescence‐like states (Wallis et al., 2022).

Chromatin reorganization and morphological alterations of the nucleus are well‐established drivers of pathology in premature aging syndromes such as the rare inherited conditions Werner syndrome (“adult progeria”) (Zhang et al., 2015) and Hutchinson‐Gilford syndrome (“child progeria”) (Goldman et al., 2004), respectively. However, it remained unclear whether and to what degree these subcellular alterations can be predictive of cell‐scale aging in contexts other than genetically determined premature aging. At the same time, distinct changes in the internal structure and shape of neutrophil nuclei have been clinically employed as diagnostic tools in microscopy‐based screenings for patients suspected of rare diseases associated with neutrophil maturation (Hoffmann et al., 2002). Utilizing a conceptually similar strategy linking nuclear form to cellular function in the context of senescence, Heckenbach et al. writing for *Nature Aging* (Heckenbach et al., 2022) described an elegant quantitative microscopy‐driven approach. This approach combines high‐content automated microscopy to capture images of thousands of nuclei and deep learning algorithms to recognize, quantify, and prioritize the nuclear morpho‐features that can distinguish between senescent and proliferating cells, thus serving as observable predictive biomarkers of cellular senescence.

The authors applied deep neural networks (DNNs) to imaging datasets obtained from control and experimentally induced senescent human primary skin fibroblasts. Trained to recognize and quantify the nuclear morphology features such as area, convexity, aspect ratio, and DAPI signal intensity (a measure of apparent DNA packaging level (Pontes et al., 2003)), DNNs were able to readily distinguish between control proliferating cells and cells undergoing passage‐mediated replicative senescence or ionizing radiation (IR)‐induced senescence. Previously reliant on subjective assessments, the automatically identified and classified as senescent nuclear alterations were calibrated against traditional molecular markers of senescence including senescence‐associated β‐galactosidase (SA‐β‐gal), p16^INK4a^, and p21^Cip1^. These and additional senescence markers confirmed the presence of senescence in cells classified as senescent by DNNs, providing validation and strengthening the reliability of the deep learning‐based classification. Experimental manipulations of senescence induction efficiency, for example, via modulation of cell population density, revealed sensitivity of DNN‐based senescence detection output to experimental senescence induction input.

Given that DNNs demonstrate a high degree of consistency in classifying senescent cells based on nuclear morphology, Heckenbach et al. asked whether their classifier can be applied to cultured primary fibroblasts from patients with Hutchinson‐Gilford progeria syndrome, ataxia telangiectasia and Cockayne syndrome as well as mouse primary astrocytes and neurons treated with IR. The authors showed that their algorithm is successful at detecting senescence across species and cell types in vitro. More excitingly, when the predictor was applied to hematoxylin and eosin (H&E)‐stained histological sections of the liver from mice of different age or human skin samples from individuals from various age groups, the algorithm quantitatively revealed increasing senescence with age. This suggested that deep learning‐based senescence detection in H&E‐stained samples can be extended to a clinical context holding promise for prognostic and diagnostic applications. Indeed, analyzing dermal biopsy images from Denmark's Pathology Specimen Biobank and linking the DNN‐evaluated senescence burden to clinical records of the biopsy donors obtained from the Danish National Patient Register from 1977 to 2018, Heckenbach et al. revealed that osteoporosis, osteoarthritis, hypertension, cerebral infarction, hyperlipidemia, hypercholesteremia, and sciatica are associated with higher levels of predicted dermal senescence, while cancer, hearing loss and dyspnea follow opposite trend. Therefore, trough the integration of clinical data and deep learning‐based senescence quantification, the authors uncovered the potential to identify senescence‐related biomarkers or signatures that predict disease progression, patient survival rates, or response to specific treatments.

This exciting cellular aging study is very much in the tradition of physics that has precision measurement at its core. The lesson that has been learned time‐and‐time‐again is that by measuring fundamental physical phenomena with increasing precision, one can make amazing discoveries and even sometimes stumble across new laws of nature. In the era of basic and clinical research dominated by molecular‐omics approaches, the idiom “seeing is believing” is becoming highly relevant. Most commonly, the identification and characterization of senescent cells relies on biochemical detection of molecular markers or often qualitative visual observations using various staining techniques. However, these approaches frequently suffer from subjectivity, variability, and limited quantification capabilities. The application of deep learning algorithms brings a transformative change by harnessing the power of visual data to detect, quantify, and classify senescent cells based on nuclear morphology. Deep learning models are trained to “see” and learn from large datasets of annotated cellular images, enabling them to recognize subtle features associated with senescence.

However, the interpretability of deep learning algorithms remains an open question and an exciting area for future research. Deep learning models are often considered black boxes due to their complex architectures and the inability to provide transparent explanations for their decisions. The lack of interpretability can hinder the understanding of the specific nuclear morphological features driving the senescence classification, limiting the ability to gain biological insights from the deep learning predictions. Heckenbach et al. made an attempt to clarify what aspects of nuclei are used by DNNs for assessment of images. They found that classification is largely based on the overall shape of nuclei. The correlation between predicted senescence and several nuclear shape descriptors established that area is moderately correlated while convexity and aspect ratio are weaker. This in turn predicted that nuclear expansion per se can be a trigger and thus an early biomarker of senescence. In order to test this prediction experimentally, we decided to inflate nuclear size by triggering whole‐genome duplication (WGD) (Gemble et al., 2022). To this end, we treated HeLa‐Kyoto cells with nanomolar concentrations of docetaxel, a semisynthetic analog of paclitaxel used as a prototypic inducer of therapy‐induced senescence (TIS) (Demaria et al., 2017). Belonging to the class of taxanes, these chemotherapeutic compounds stabilize cytoskeletal microtubules, which interferes with vesicular trafficking in interphase cells (Lomakin et al., 2009) and the assembly and functioning of the spindle apparatus in mitotic cells (Yvon et al., 1999). Blocked in mitosis, taxane‐treated cells undergo apoptosis via mitotic catastrophe (Weaver, 2014). However, some mitotically arrested cells in the population can spontaneously evade this program and go through the so‐called mitotic slippage, resulting in an interphase cell whose DNA content was not partitioned in the previous mitotic phase (Weaver, 2014). Such interphase cells are also prone to DNA endoreplication during which the cell cycles between S‐ and G‐phases of the cell cycle entirely skipping the M phase (Shu et al., 2018). The net effect from these perturbations is nuclear expansion due to polyploidy‐mediated DNA content increase (Figure 2a). Because taxane treatment results in a substantial cell death often leaving even at a low magnification only a few cells per field of view, we applied high‐content automated microscopy to capture as many as possible images of nuclei. Employing machine learning‐based tools to segment the images and extract nuclear morphological features driving the senescence classification established in Heckenbach et al., we were able to detect a significant change of all key nuclear morphometric descriptors predictive of senescence as early as 3 days after taxane exposure (Figure 2b). Consistent with the nuclear morphology‐based senescence prediction, the cells exhibited yet another morphological hallmark of senescence—cell size increase (Figure 2c), which was highly correlated with nuclear size increase (Pearson *r* = 0.82, *p* < 0.0001). Additionally, our flow cytometry‐based measurements showed that the cells possess significantly increased cytoplasmic granularity (Figure 2c), which is a defining feature associated with organellar and metabolic alterations in senescent cells (Gorgoulis et al., 2019).

**FIGURE 2:**
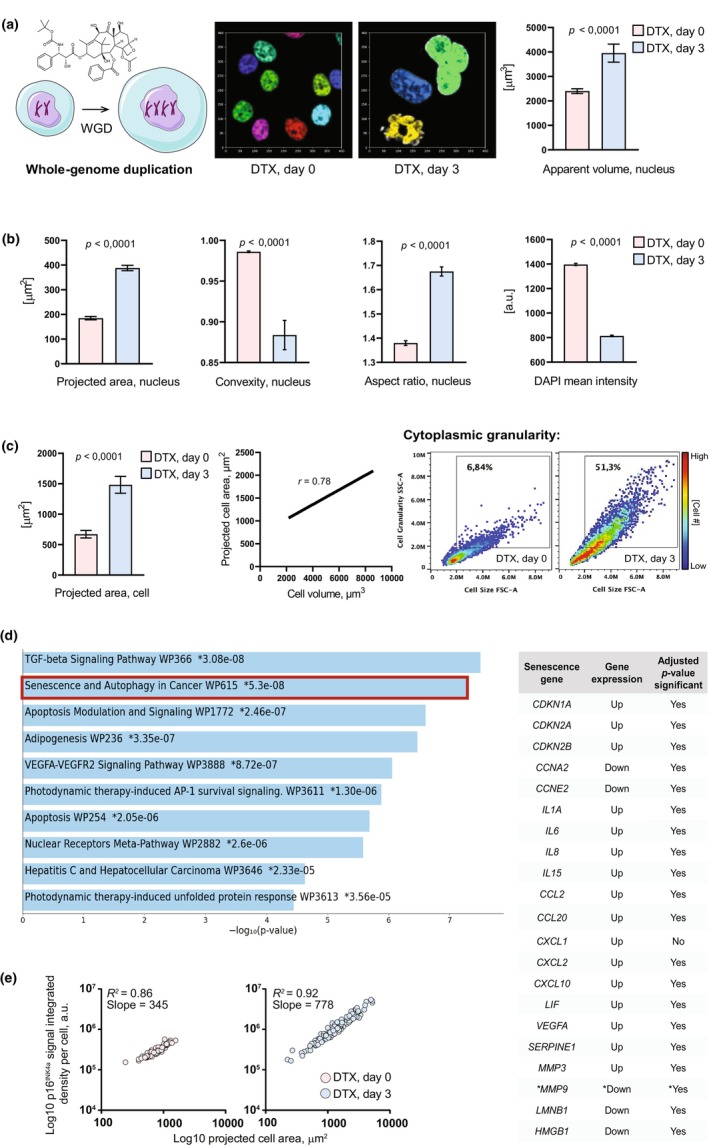
Experimental manipulations of nuclear morphology via cell ploidy increase trigger a senescence(‐like) state: proof‐of‐principle study. (a) Exposure of HeLa‐Kyoto cells to the taxane chemotherapy agent docetaxel (DTX) induces nuclear expansion due to whole‐genome duplication (WGD). Apparent nuclear volume data, mean ± SEM, *n* = 100 cells per each condition; *p*‐value, unpaired two‐tailed *t* test. (b) Polyploid HeLa‐Kyoto cells begin exhibiting the nuclear morphology features associated with senescence, established by Heckenbach et al., 2022, as early as 3 days of exposure to DTX coincident with the altered nuclear size. Mean ± SEM, *n* > 250 cells per each condition; *p*‐value, unpaired two‐tailed *t* test. (c) Paralleled by nuclear expansion, whole‐cell size increases in polyploid cells as evident by the increase in projected cell area (mean ± SEM, *n* > 250 cells per each condition; *p*‐value, unpaired two‐tailed *t* test) that can serve as a proxy for cell size because it is highly correlated with cell volume (*r*, Pearson correlation coefficient; *p* = 0.0079, unpaired two‐tailed *t* test). Whole‐cell size increase is accompanied by an increase in cytoplasmic granularity as follows from FACS‐based side scatter readouts (SSC‐A ordinate) against cellular size (FSC‐A abscissa) (*n* = 10,000 cells per each condition, *p* < 0.0001). (d) Left, RNA‐Seq of polyploid cells reveals senescence as a top transcriptional signature as early as 3 days of exposure to DTX coincident with altered nuclear and cell size. Right, almost all (except *MMP9* marked with a star sign) key senescence‐associated transcripts exhibit expected up‐ or downregulation in polyploid cells. (e) Cell size‐dependent increase in the expression of p16^Ink4a^ (*n* = 100 cells per each condition), a senescence‐associated protein marker whose transcript *CDKN2A* gets upregulated as follows from the RNA‐Seq of polyploid cells.

We then decided to investigate whether our morphology‐based classification of senescence is consistent with senescence induction at the molecular level. To this end, we performed RNA‐Seq of cells on Day 0 versus Day 3 of taxane exposure and comparatively analyzed their transcriptomic landscape. Gene set enrichment analysis (GSEA) of the transcripts whose expression was upregulated in a statistically significant fashion revealed “Senescence & autophagy in cancer” as a top transcriptional signature of cells after 3 days of taxane treatment (Figure 2d). We then interrogated our transcriptomic dataset and compared it against published transcriptional signatures associated with cellular senescence (Gonzalez‐Gualda et al., 2021; Gorgoulis et al., 2019). Remarkably, almost all transcripts typically associated with senescence were consistently regulated in a statistically significant manner in our experimental system (Figure 2d), thus confirming the induction of senescence revealed by nuclear morphology markers. Indeed, we were able to detect significant upregulation of the senescence‐associated cell cycle inhibitor p16^INK4a^ protein, whose increased expression was highly correlated with increasing cell size (Figure 2e). Finally, consistent with rather early manifestations of senescence at the molecular and nuclear morphology levels, we found that SA‐β‐gal fluorescent probe signal gets upregulated on Day 3 of taxane exposure (26.54 ± 0.62 a.u./cell on Day 0 vs. 49.29 ± 2.10 a.u./cell on Day 3; data, mean ± SEM, *p* < 0.0001), and this upregulation increases further (49.29 ± 2.10 a.u./cell on Day 3 of DTX exposure vs. 59.76 ± 3.78 3 days after DTX washout; data, mean ± SEM, *p* < 0.05) after additional 3 days upon taxane therapy washout. This suggests that nuclear and cell morphology changes precede the development of a more advanced senescence state thus exposing the predictive power of (sub)cellular morphology in detecting cells committing to a senescence fate. We believe that this is a universal phenomenon because we can reproduce nuclear morphology alterations predictive of senescence in an independent human cancer cell line DU145 (prostate carcinoma, data not shown).

One challenge that remains is understanding how the findings from in vitro experiments apply to complex 3D tissue environments. In their analysis of H&E‐stained mouse and human biopsy samples, Heckenbach et al. observed either a negligible increase or no significant increase in nuclear area with age. This suggests the possibility of distinctions in how senescence manifests or the specific mechanisms involved when comparing cells cultured in 2D to cells within 3D tissue sections. However, we propose that this apparent discrepancy may be attributed to the need for a precise assessment of nuclear size within the context of 3D tissues, which necessitates the reconstruction of nuclear volume or the examination of nuclear boundaries at various depths along the tissue Z‐axis (the vertical axis). Relying solely on a single cross‐section of tissue, where cell nuclei are situated at different depths along the Z‐axis, may not accurately reflect potential variations in nuclear size. Exploring this issue further represents an avenue for future research studies.

Overall, Heckenbach et al. and our own confirmatory yet complementary study briefly presented here demonstrate the potential of nuclear morphology as a robust and reliable biomarker of senescence. The identified nuclear alterations, including changes in nuclear size, shape, and texture, provide quantitative and objective measures to predict cellular senescence. The mechanistic understanding of the causal link between a quantifiable change in nuclear morphology (e.g., size) per se and the induction of senescence is an exciting question for future research in the field of basic cell biology. The ability to accurately detect and quantify cellular senescence based on nuclear morphology has broad implications for multiple biomedical fields, including aging and cancer research, regenerative medicine, and drug discovery. The deep learning‐based biomarker has the potential to revolutionize the development of diagnostic assays, enabling early detection of senescent cells in tissues or biofluids. Additionally, it offers a powerful tool for assessing the effectiveness of antiaging interventions and potential therapeutic compounds in preclinical and clinical studies.

## AUTHOR CONTRIBUTIONS

Alexis J. Lomakin conceived the idea for this study and developed the overall study design. Jakub Belhadj and Surina Surina performed the experiments, analyzed the data, and drafted the manuscript with input from Markus Hengstschläger and Alexis J. Lomakin. All authors edited and approved the final manuscript.

## FUNDING INFORMATION

S.S. was supported by an international Ph.D. scholarship from the University of Campania Luigi Vanvitelli. A.J.L. was supported by the Fellinger Krebsforschung (grant SENSECARE 2023‐07‐0046) and internal departmental funds of the Center for Pathobiochemistry & Genetics, Medical University of Vienna.

## CONFLICT OF INTEREST STATEMENT

None declared.

## Supporting information


Supporting Information

## Data Availability

Data sharing is not applicable to this article as no new data were created or analyzed in this study.
