# Correction to “Form Follows Function: Nuclear Morphology as a Quantifiable Predictor of Cellular Senescence”

**DOI:** 10.1111/acel.70076

**Published:** 2025-04-16

Belhadj J, Surina S, Hengstschläger M, Lomakin AJ. Form follows function: Nuclear morphology as a quantifiable predictor of cellular senescence. *Aging Cell*. 2023 Dec;22(12):e14012.

The “OPEN RESEARCH” section of the article did not include information regarding the RNA‐seq data and its availability to the wider community. We would like to correct this omission by updating the “DATA AVAILABILITY STATEMENT” in the “OPEN RESEARCH” section with the following:

“The raw data for the RNA‐seq analysis used in this study have been deposited in the NCBI GEO public database under accession number GSE293637 (https://www.ncbi.nlm.nih.gov/geo/query/acc.cgi?acc=GSE293637).”

We apologize for this omission.

