# Supplementary material for: Form follows function: Nuclear morphology as a quantifiable predictor of cellular senescence
Source: Aging Cell. 2023 Oct 16;22(12):e14012. doi: 10.1111/acel.14012 (PMC10726876; doi:10.1111/acel.14012)
Supplement: Supplementary file 1 — Supporting Information [file ACEL-22-e14012-s001.docx]

**Materials and methods**

**Cell culture**

HeLa Kyoto cells were cultured in Dulbecco's Modified Eagle Medium-Nutrient Mixture F-12 (DMEM-F12) medium (11330032, Thermo Fisher Scientific, US), supplemented with 10% fetal bovine serum (FBS) and 1% penicillin/streptomycin. The cell cultures were maintained in humidified incubators at 37 °C with 5% CO_2_. In subsequent experiments, HeLa Kyoto cells were exposed to 10 nM Docetaxel (DTX) for 72 hours under standard cell culture conditions.

**Cell size and volume measurements**

After Docetaxel treatment, HeLa Kyoto cells were washed two times with warm (37°C) PBS (D8662, Sigma/Merck). Afterwards, cells were fixed with warm (37°C) 4% PFA (28908, Thermo Scientific™) for 10 minutes, then washed three times with PBS for 5 minutes each on a shaker. Next, cell nuclei were stained with Hoechst 33342 (R37605, Invitrogen NucBlue™ Live ReadyProbes™). To reveal cell boundaries, F-actin was stained with phalloidin conjugated with a green-fluorescent dye (ActinGreen™ 488 ReadyProbes™, R37110, Invitrogen). Protected from light, the samples were incubated for 30 minutes on a shaker and subsequently washed with PBS three times as previously for 5 minutes each. Samples were mounted on microscope slides (S8902, Sigma/Merck) with ProLong Glass Antifade Mountant (P36980, Thermo Fischer) and imaged using EVOS™ M7000 Imaging System (AMF7000, Invitrogen) with 40x objective (Olympus™ 40X Oil Objective, X-Apo, 1.40NA/0.13WD). Fluorescence emitted by Hoechst 33342 was detected using the DAPI light cube (AMEP4950, Invitrogen); the signal emitted by phalloidin conjugated with ActinGreen™ 488 dye was detected with the GFP light cube (AMEP4951, Invitrogen). Automated quantitative analysis of nuclear morphometric features and cell size measurements were performed using Celleste 6.0™ software (AMEP4942, Invitrogen).

**Immunofluorescent staining**

HeLa Kyoto cells were stained with NucBlue live cell stain( R37605, Thermo Fisher Scientific,US) for 45 min in a humidified incubator at 37 °C with 5% CO2. After this step, the cells were fixed with 4% paraformaldehyde for 15 minutes and permeabilized with 0.5% Triton-X 100 for 5 minutes at room temperature. The cells were then incubated for 1 hour with primary antibodies against p16^Ink4^ (ZRB1437, Merck Group, Germany) at a dilution of 1:200. Following this step, the cells were washed five times with PBS for 5 minutes each on a shaker and subsequently incubated for 1 hour with Alexa Fluor 488 conjugated secondary antibodies (A11008, Thermo Fisher Scientific, US) at a dilution of 1:300 at room temperature. Microscopy samples were mounted and imaged as described in the subsection “Cell size and volume measurements”.

**RNA isolation and bulk RNA sequencing**

HeLa Kyoto cells treated with 10nM Docetaxel for 72 hours (DTX, day 3) and control cells from the same cell line (DTX, day 0) were prepared in triplicates. The cells were directly lysed/detached with RLT Buffer (NC9497582, Qiagen) substituted with 100x dilluted β-mercaptoethanol. RNA was extracted using the RNeasy Micro Kit according to manufacturer´s instructions (74004, Qiagen). Bulk RNA sequencing (stranded mRNA-seq (poly-A enrichment)) and bioinformatics analysis were performed by the Biomedical Sequencing Facility of the CeMM - Research Center for Molecular Medicine of the Austrian Academy of Sciences (Vienna, Austria).

**Flow cytometry**

Following Docetaxel (DTX) treatment, the cells were detached using trypsin and collected into 1.5 mL tubes. The cell suspension was then subjected to centrifugation at room temperature for 3 minutes at 900 rpm. Cells were then washed once with phosphate-buffered saline (PBS). The supernatant was carefully discarded, and the cells were resuspended in PBS. Subsequently, the relative cell size and granularity were assessed using flow cytometer (CytoFLEX S, Beckman Coulter, US).

**Statistic analysis**

Each experiment was carried out as three independent repeats. 100-500 cells were quantified in experiments requiring image analyses. All statistical analyses were performed with GraphPad Prism 9 software. Data are presented as mean ± SD. Statistical differences were determined by unpaired two-tailed *t* test.
